# In Memoriam: V. Reggie Edgerton

**DOI:** 10.1186/s12984-026-02152-x

**Published:** 2026-09-29

**Authors:** Richard L. Lieber, David J. Reinkensmeyer, Joel W. Burdick, Arun Jayaraman

**Affiliations:** 1 California Institute of Technology, Pasadena, California, USA; 2 Shirley Ryan AbilityLab and Northwestern University, Chicago, Illinois USA; 3 University of California, Irvine, California, USA

Victor R. Edgerton passed away at the age of 85 on Wednesday, March 25, 2026. Reggie, as he was known by his colleagues, transformed rehabilitation through his innovative work in neurophysiology and rehabilitation engineering. While he was an exercise scientist by training, Reggie was an incredibly influential physiologist and rehabilitation engineer. His work parsing the foundational physiological mechanisms that drive movement shaped the field, and it served as a springboard for his translational work in developing groundbreaking therapeutic tools that are still being studied and utilized today. Over the course of his productive career, he helped usher in a “new age” of rehabilitation that combined neuromodulation and robotic training as a therapeutic strategy [4].

## Engineering rehabilitation around locomotion

One of Reggie’s most influential contributions was his work developing systems to facilitate locomotor training after spinal cord injury. Grounded in his basic physiology studies, Reggie recognized that simply engaging the somatosensors and muscles involved in locomotion by manually moving limbs could deliver nuanced, contextualized sensory information to spinal networks and effectively “re-train” them to generate the necessary signals to their downstream muscle groups [11, 14]. Reggie liked to say that the spinal cord is analogous to the brain, rather than a cable, because it is capable of both sophisticated sensory processing and motor learning [3]. He leveraged these insights to develop devices that would support this type of training in a controlled, quantifiable, and easily modifiable way. In 1998, Reggie and his team were manually training spinal-transected rats to step using cotton swabs when they enthusiastically embraced a proposal to automate the training using robotics, sparking a life-long commitment to robotic rehabilitation. The result was the “rat stepper,” a robotic device that facilitated patterned stepping in animals with a complete spinal cord lesion [16]. This work and the further robotic developments and scientific insights it ultimately supported, including the importance of variability produced by assistance-as-needed in locomotor training [2, 21], highlight the translational nature of Reggie’s thinking. While robotics were a boon for basic scientists, giving them a new tool to use for preclinical rodent studies, it also served as a steppingstone to the creation of similar tools for therapeutic use in patients. Reggie supported the development of a variety of human gait-training robotic systems throughout his career [18, 5, 22], eventually publishing a seminal case study on the implementation of an exoskeleton to treat a patient with chronic paralysis [8]. Combined with other interventions, which were also the product of Reggie’s foundational work, the use of this exoskeleton for locomotor training helped improve the patient’s voluntary motor control. This marked a major breakthrough for the field and was part of a paradigm shift in neurorehabilitation, which was achieved largely because of Reggie’s persistent efforts.

## Neuromodulation as an enabling technology

While Reggie’s foundational studies focused on the use of training to promote plasticity in spinal networks, his later work showed that direct stimulation could enhance this therapeutic goal. His early engineering efforts centered on epidural stimulation, where implanted electrodes in rats made it possible to test how specific stimulation parameters paired with targeted training could influence stepping behavior and load bearing [9, 6, 7, 15]. These preclinical systems provided an essential bridge to human translation. In landmark clinical studies, Reggie’s team paired epidural stimulation with task-specific training, which they showed could enable SCI patients to recover some of their voluntary lower-limb movement [1, 12]. Reggie and his collaborators also extended this strategy to cervical networks, helping show that neuromodulation could support recovery of hand and upper-extremity function as well [13, 17].

Reggie was also keenly aware that implanted systems had limitations that could hinder their implementation, which inspired him to also study transcutaneous spinal stimulation as a more scalable and less invasive alternative. His work showed that stimulation delivered through the skin could still modulate spinal excitability and support motor recovery. He also worked diligently to fine-tune these approaches, finding that stimulation delivered across multiple sites rather than a single site could offer more consistent and robust improvements [10, 19, 20].

## A lasting impact on rehabilitation engineering

It should thus be obvious that modern, activity-based rehabilitation, especially programs that combine robot-assisted training with neuromodulation, exist in large part due to Reggie’s conviction that therapeutic technology must be developed based on systematically and rigorously proven neurophysiological principles, including the fundamental observation of a smart, trainable, spinal cord. This integrated view of physiology, engineering, and clinical care were a hallmark of Reggie’s scientific career.

Reggie has left an indelible imprint on the fields of physiology, engineering, and rehabilitation. He showed that rehabilitation technologies are not limited to assistive functions, but rather they can be used as therapeutic tools to engage physiological mechanisms and provide true and lasting recovery. Through this work, he reshaped the field of neurorehabilitation, and the field is more ambitious because of him. It is also more willing to build technologies that respect the intelligence of the nervous system, especially the spinal cord. Those who continue to develop locomotor training systems, neuromodulation devices, and activity-based therapies are forever indebted to Reggie for his foundational and groundbreaking work that set the stage for these emerging technologies. He will be deeply missed, and we will be forever grateful for him.
